# Supplementary figures and images for: Myristoylated Neuronal Calcium Sensor-1 captures the preciliary vesicle at distal appendages
Source: eLife. 2025 Jan 30;14:e85998. doi: 10.7554/eLife.85998 (PMC11984960; doi:10.7554/eLife.85998)

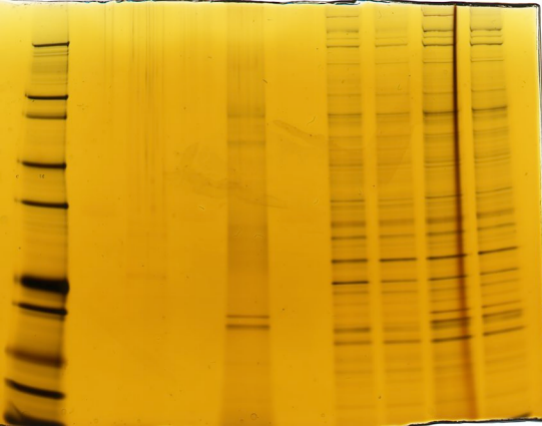

Supplement: Figure 1—source data 1. [file elife-85998-fig1-data1.pdf]

Figure 1D\_GFP

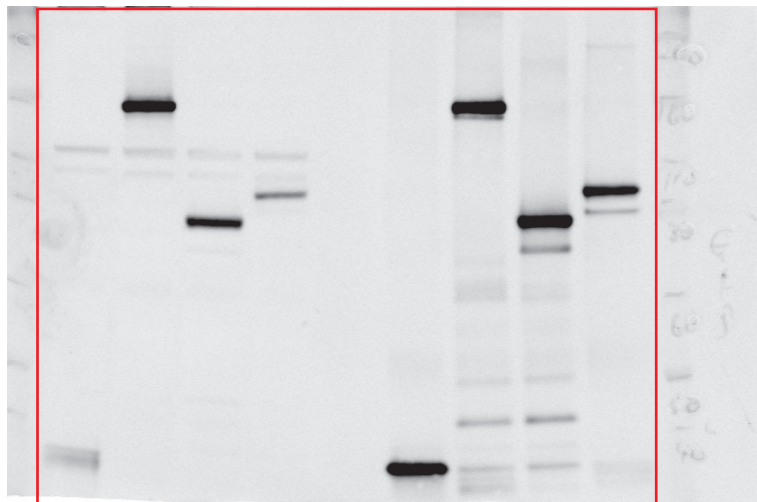

Figure 1D\_NCS1

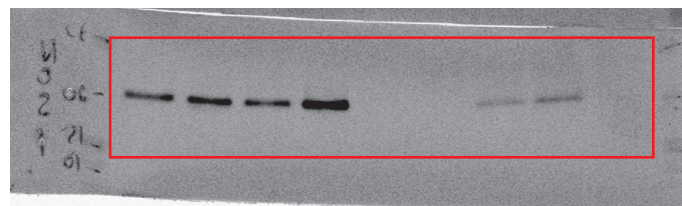

Figure 1D\_Tubulin

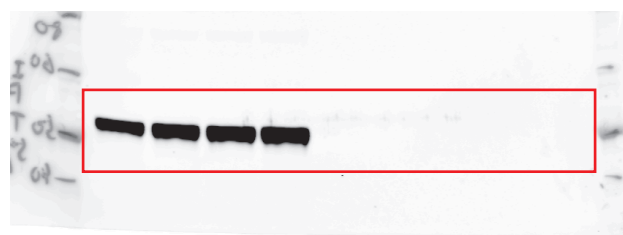

Supplement: Figure 1—source data 4. [file elife-85998-fig1-data4.pdf]

Figure 1E\_anti-MYC

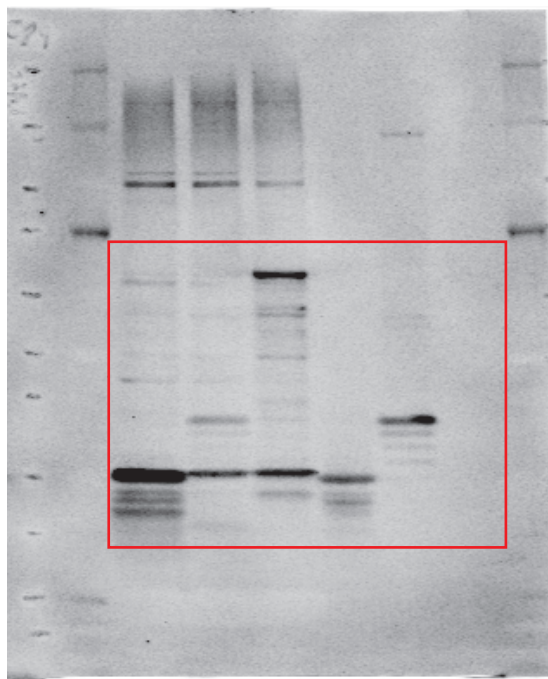

Figure 1E\_anti-HA

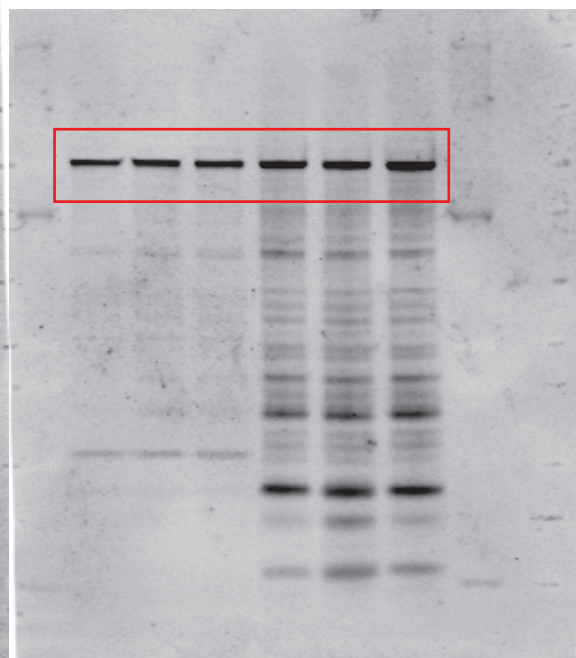

Supplement: Figure 1—source data 6. [file elife-85998-fig1-data6.pdf]

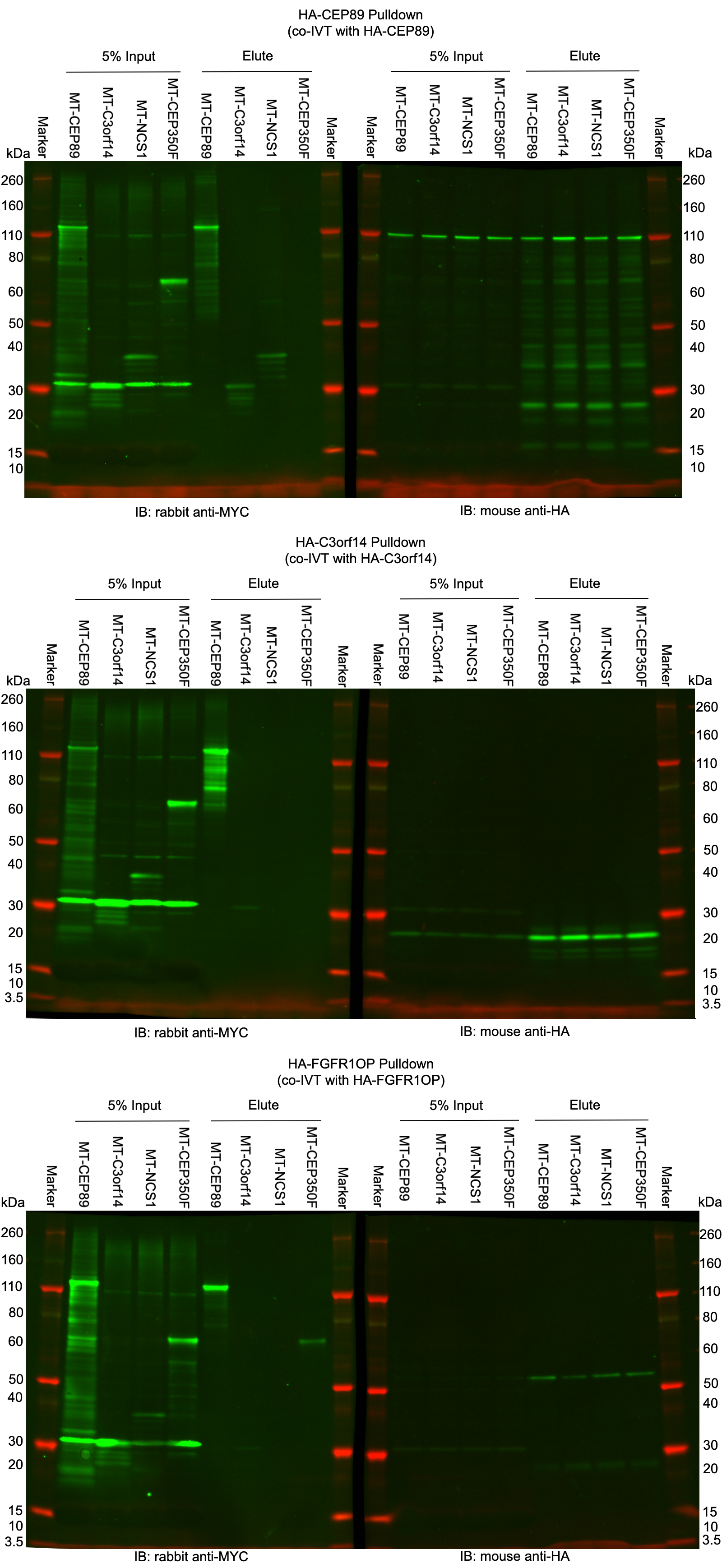

Supplement: Figure 1—source data 7. [file elife-85998-fig1-data7.zip › Figure 1-Source Data 7/2021-11-17 Replicate 1/2021-11-12/2021-11-12 Pulldowns.png]

Figure 1F\_anti-MYC

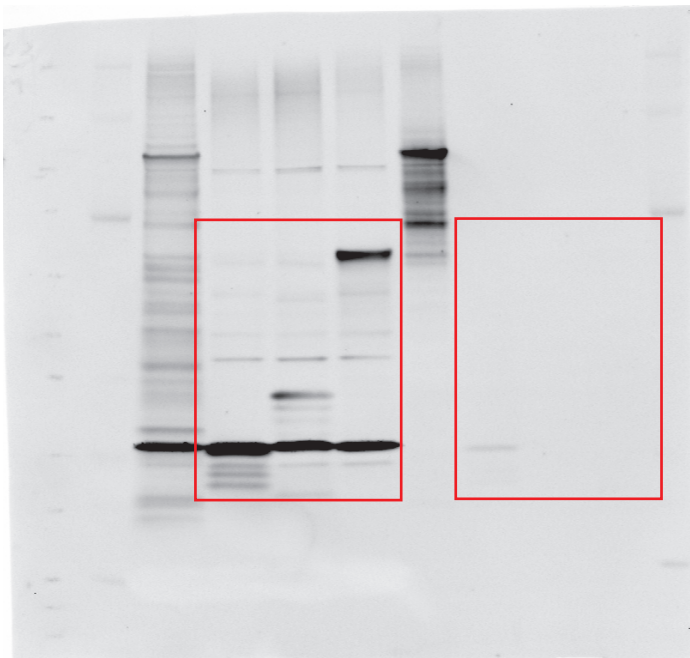

Figure 1F\_anti-HA

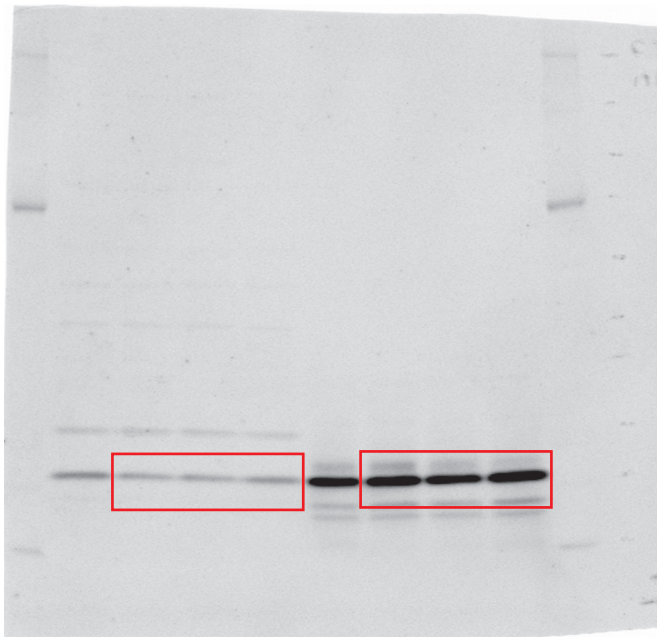

Supplement: Figure 1—source data 8. [file elife-85998-fig1-data8.pdf]

Figure 1-figure supplement1A\_CEP89

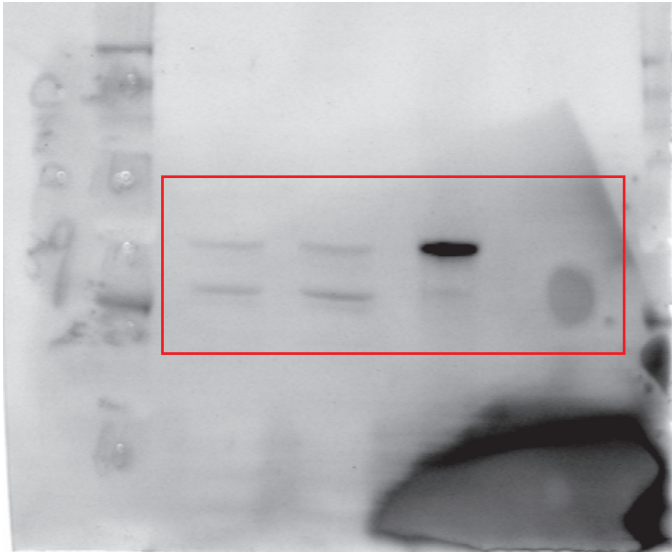

Figure 1-figure supplement1A\_NCS1

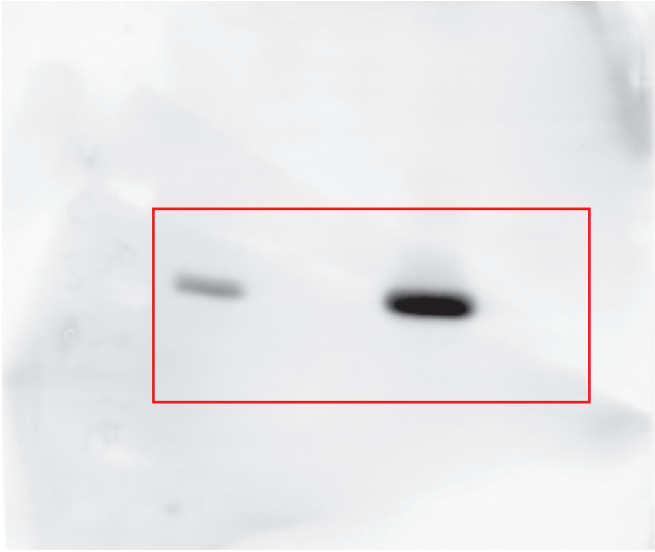

Supplement: Figure 1—figure supplement 1—source data 2. [file elife-85998-fig1-figsupp1-data2.pdf]

Figure 1-figure supplement 2A\_MYC

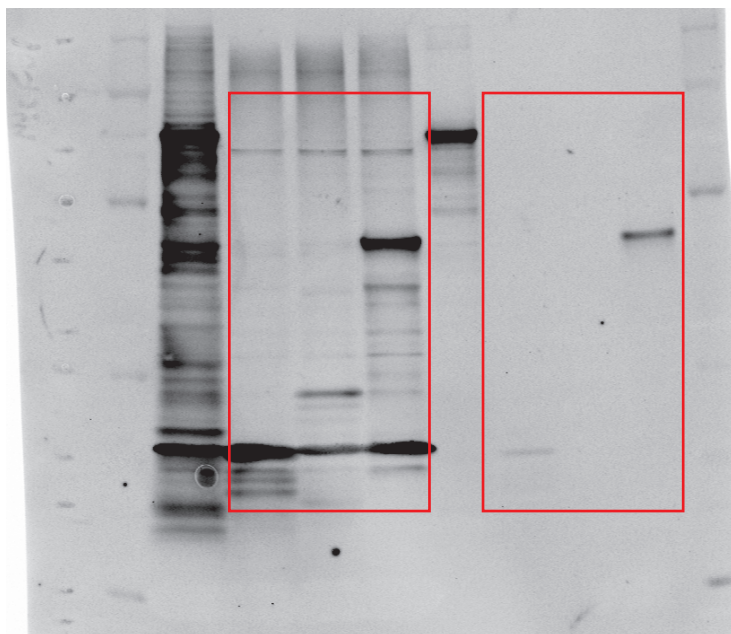

Figure 1-figure supplement 2A\_HA

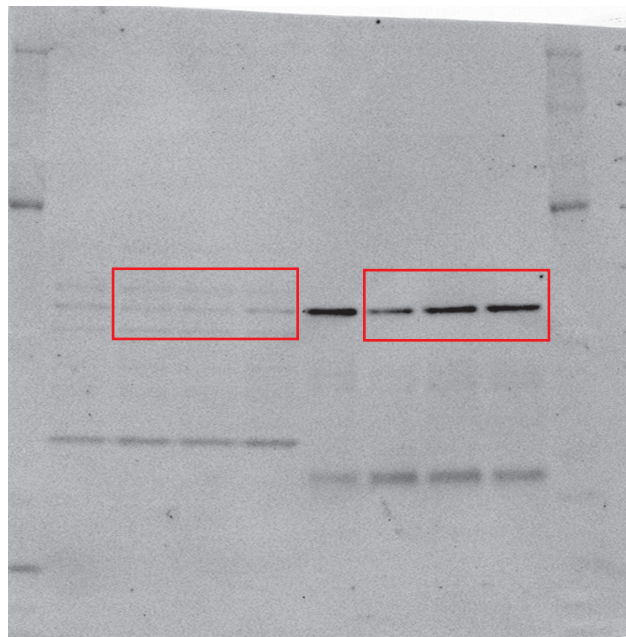

Supplement: Figure 1—figure supplement 2—source data 2. [file elife-85998-fig1-figsupp2-data2.pdf]

Figure 2-figure supplement 2E\_anti-GFP

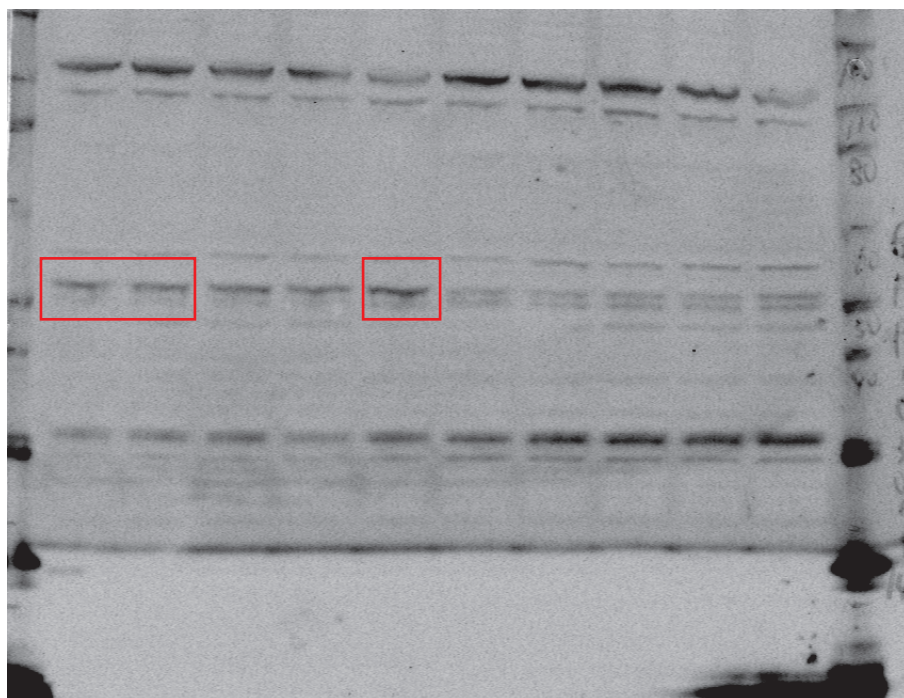

Figure 2-figure supplement 2E\_anti-Tubulin

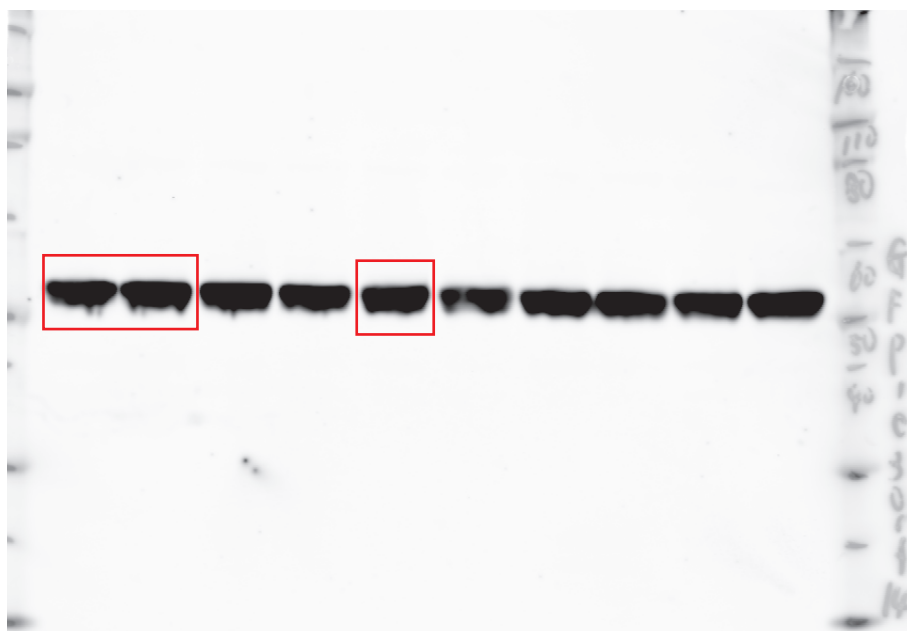

Supplement: Figure 2—figure supplement 2—source data 7. [file elife-85998-fig2-figsupp2-data7.pdf]

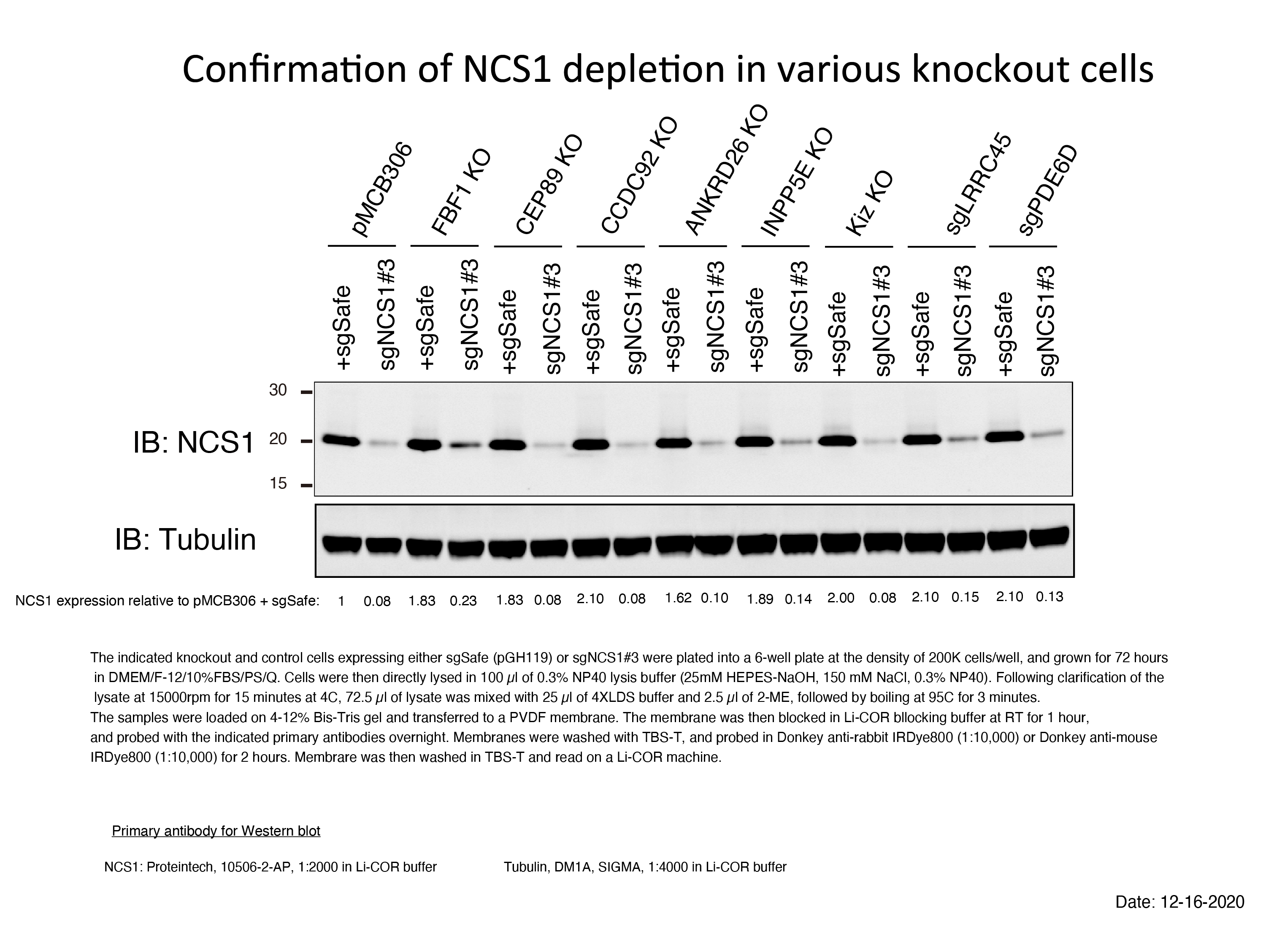

Supplement: Figure 4—source data 1. [file elife-85998-fig4-data1.zip › Figure 4-Source Data 1/KANW189_sgNCS1.jpg]

Figure 4A\_anti-NCS1

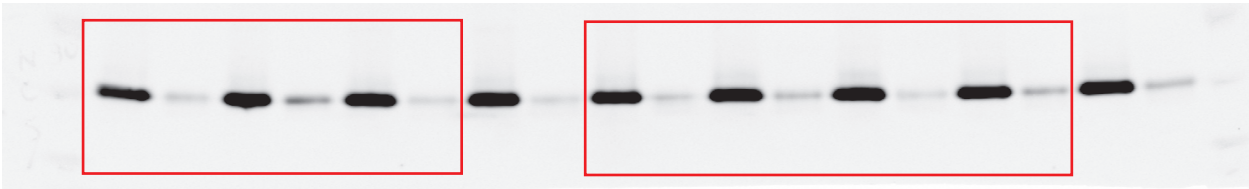

Figure 4A\_anti-Tubulin

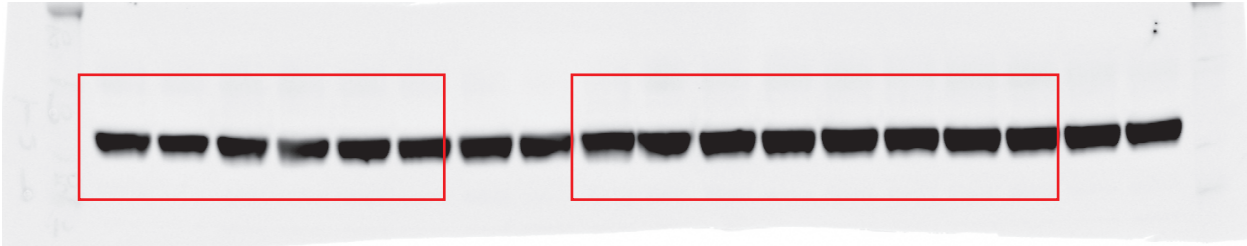

Supplement: Figure 4—source data 2. [file elife-85998-fig4-data2.pdf]

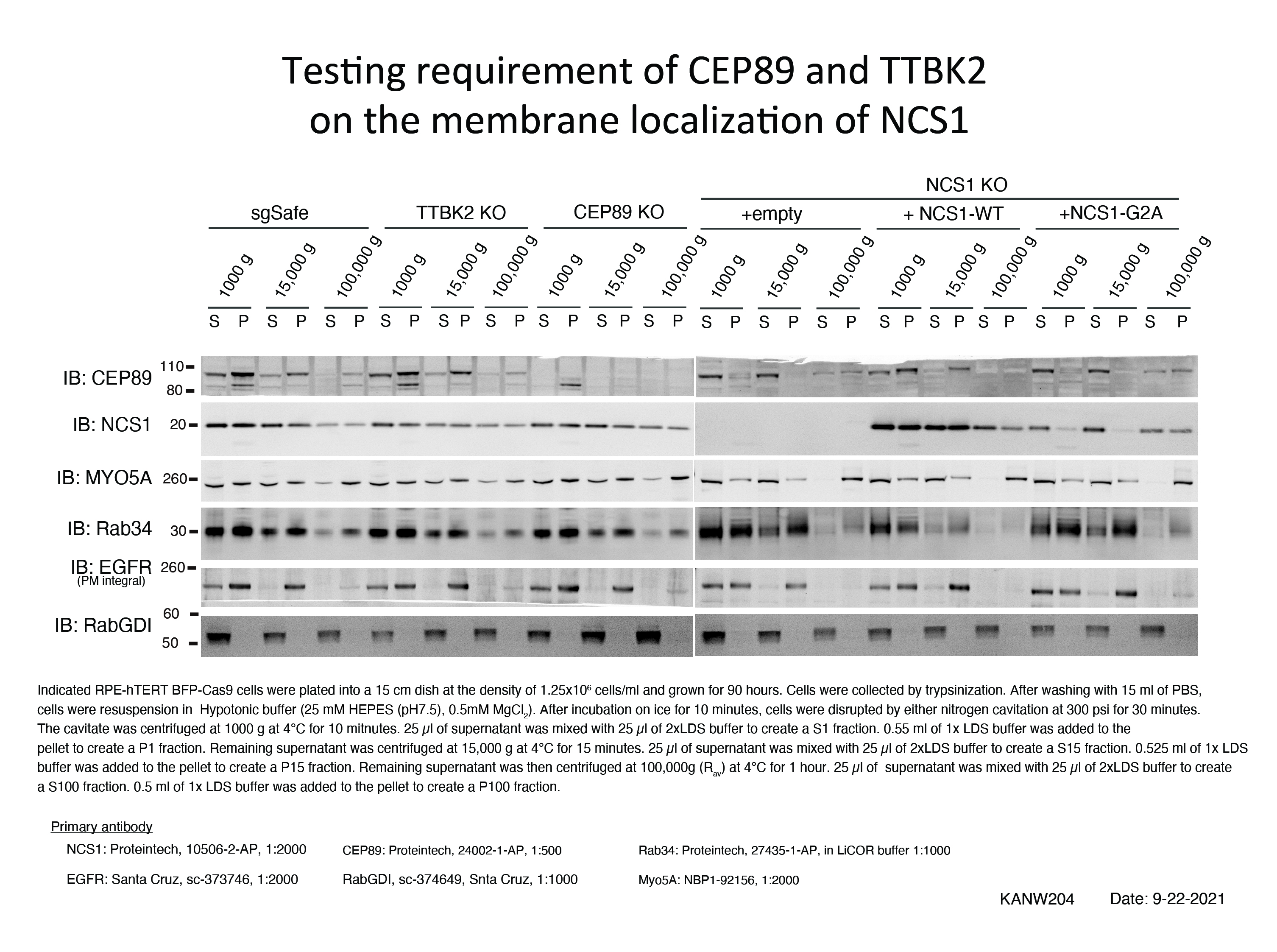

Supplement: Figure 5—source data 1. [file elife-85998-fig5-data1.zip › Figure 5-Source Data 1/KANW204_Optimization of homogenization.jpg]

Figure 5A\_CEP89

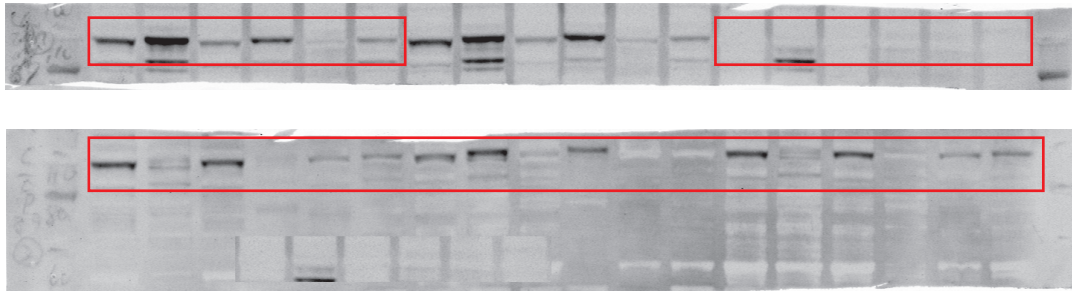

Figure 5A\_NCS1

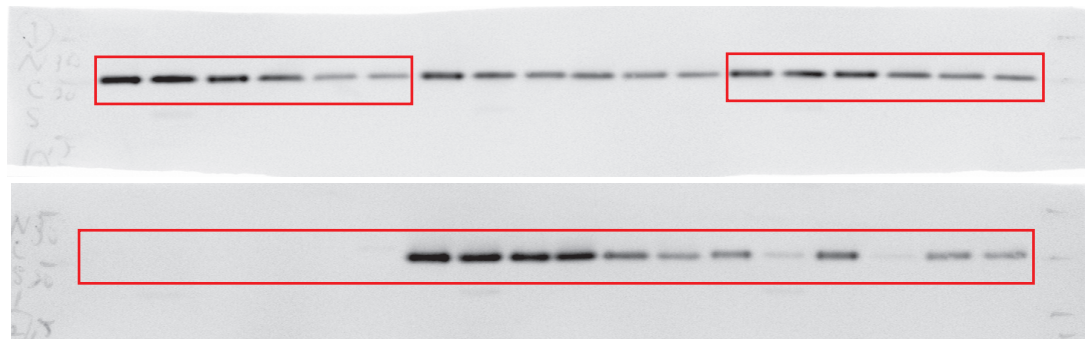

Figure 5A\_EGFR

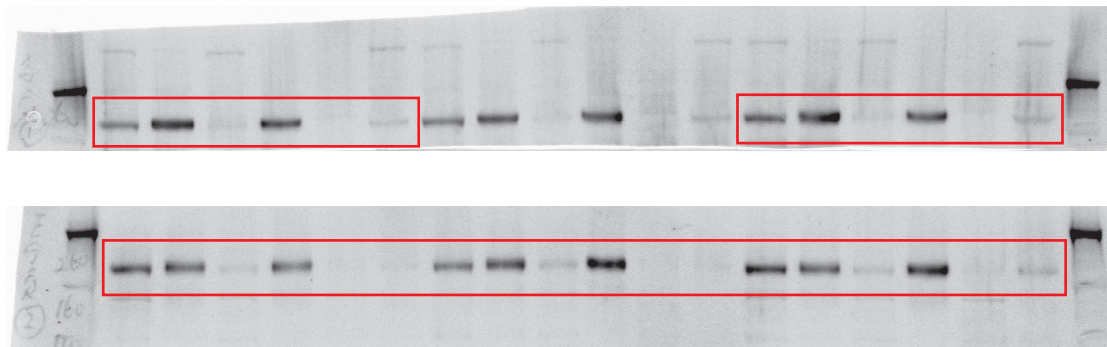

Figure 5A\_RabGDI

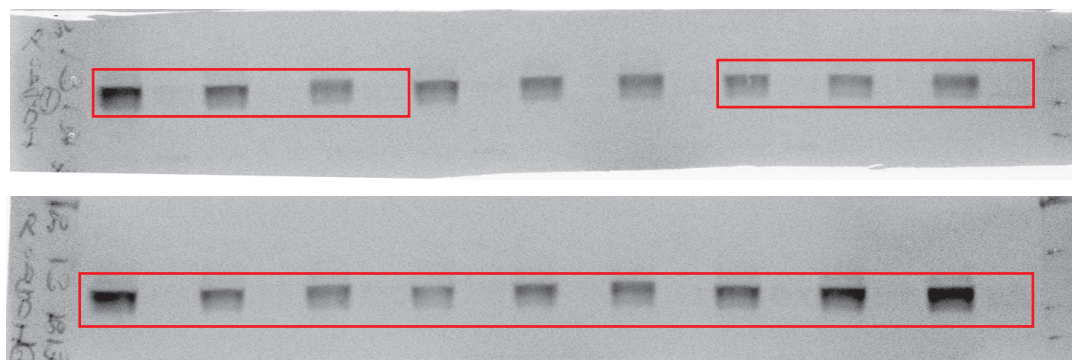

Supplement: Figure 5—source data 2. [file elife-85998-fig5-data2.pdf]

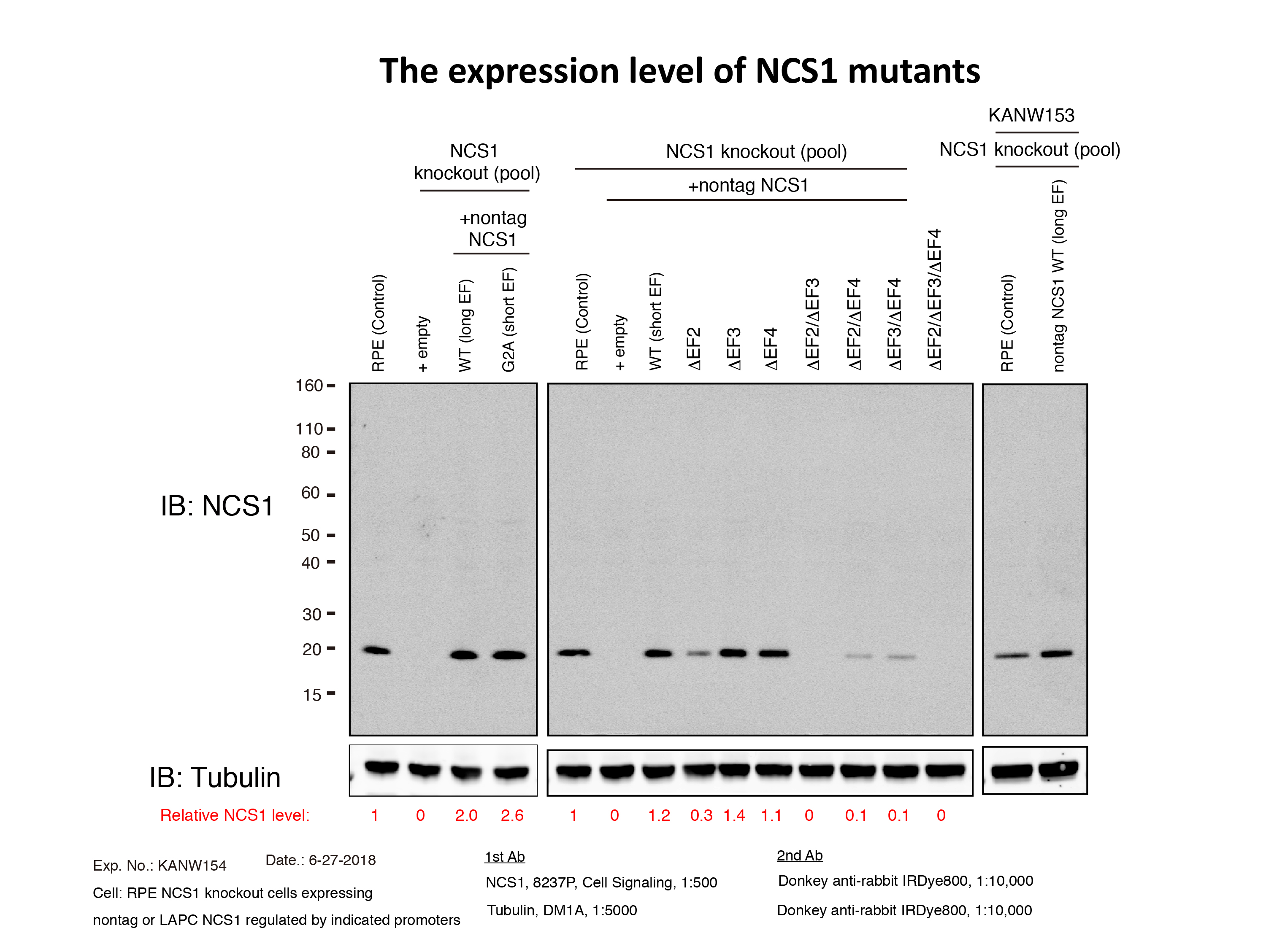

Supplement: Figure 5—source data 3. [file elife-85998-fig5-data3.zip › Figure 5-Source Data 3/KANW154/KANW154_Expression level of NCS1 mutants.jpg]

Figure 5B\_NCS1

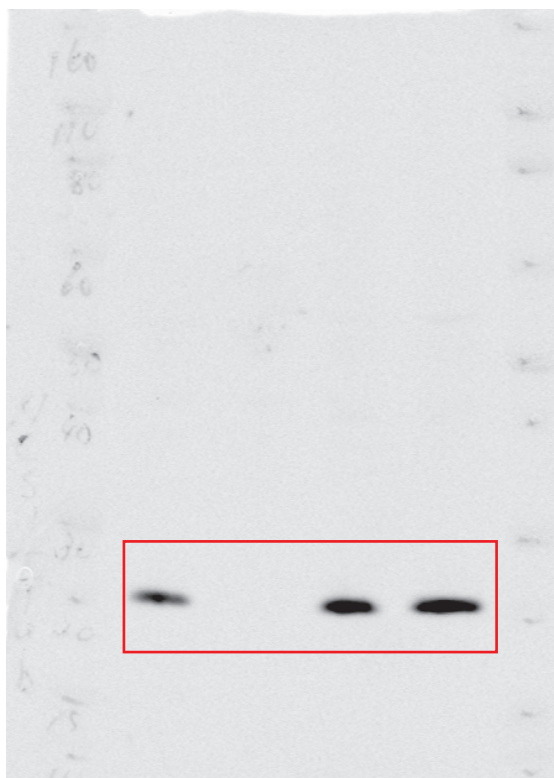

Figure 5B\_Tubulin

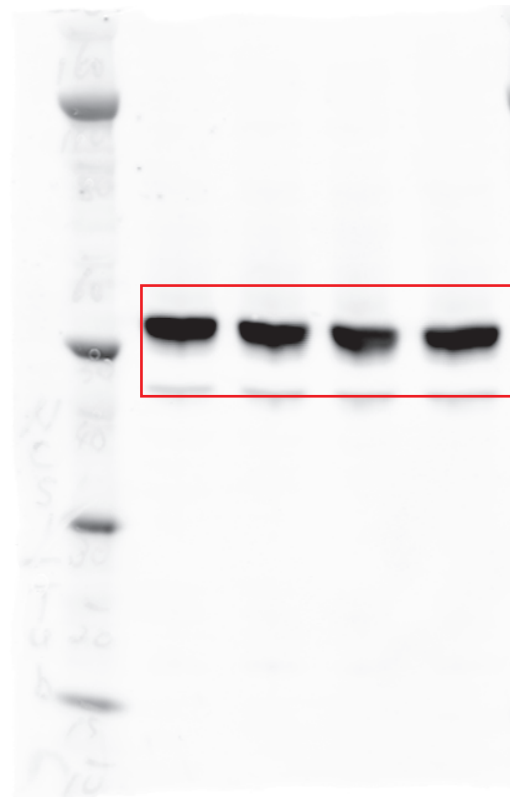

Supplement: Figure 5—source data 4. [file elife-85998-fig5-data4.pdf]

Figure 5-figure supplement 1A\_GFP (CEP89)

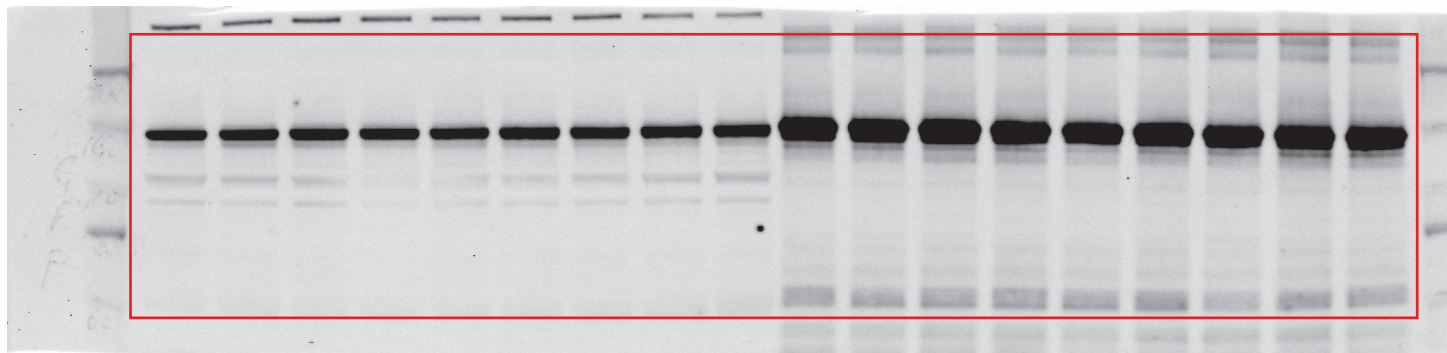

Figure 5-figure supplement 1A\_NCS1

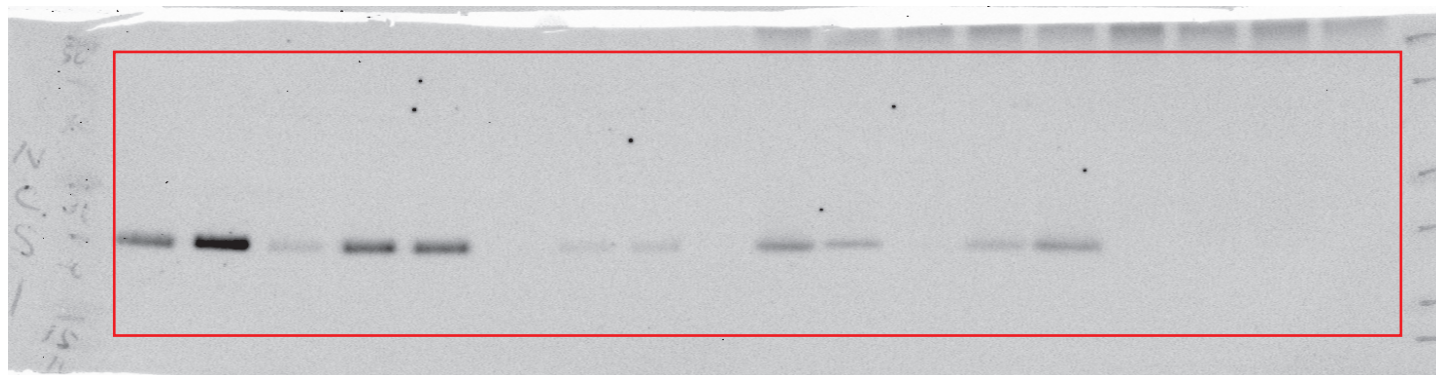

Supplement: Figure 5—figure supplement 1—source data 2. [file elife-85998-fig5-figsupp1-data2.pdf]
