# Supplementary material for: Myristoylated Neuronal Calcium Sensor-1 captures the preciliary vesicle at distal appendages
Source: eLife. 2025 Jan 30;14:e85998. doi: 10.7554/eLife.85998 (PMC11984960; doi:10.7554/eLife.85998)
Supplement: Figure 2—figure supplement 2—source data 5. [file elife-85998-fig2-figsupp2-data5.pdf]

Figure 2-figure supplement 2D_anti-CEP89

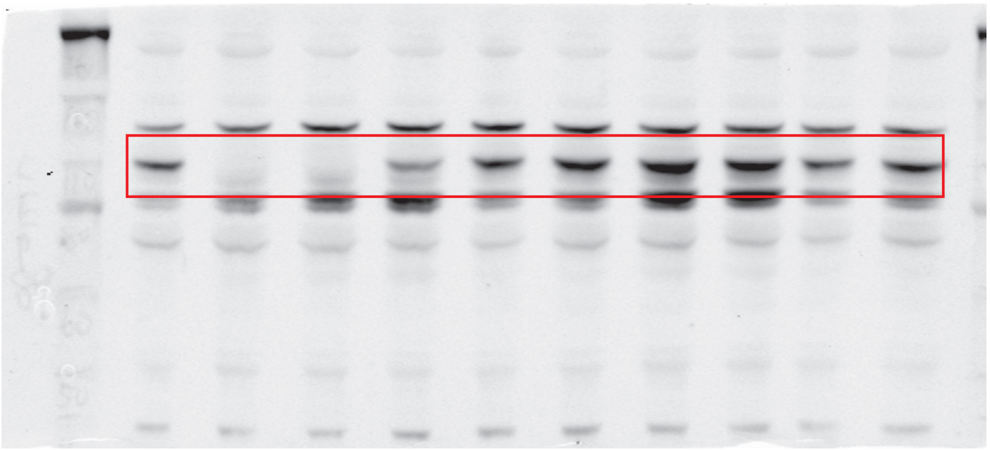

Figure 2-figure supplement 2D_anti-NCS1

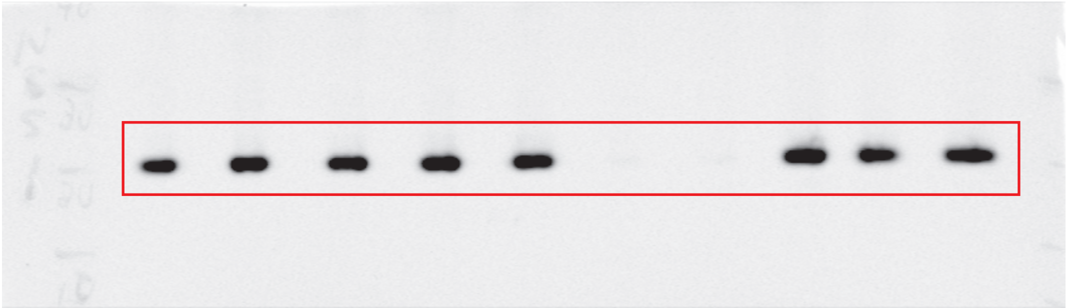

Figure 2-figure supplement 2D_anti-Tubulin

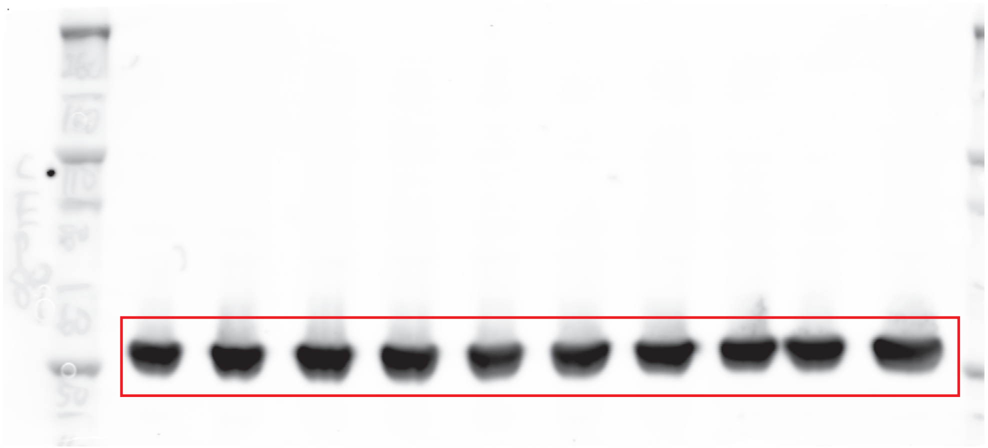

Kanie et al., Figure 2-figure supplement 2-Source Data 5
