# Supplementary material for: Myristoylated Neuronal Calcium Sensor-1 captures the preciliary vesicle at distal appendages
Source: eLife. 2025 Jan 30;14:e85998. doi: 10.7554/eLife.85998 (PMC11984960; doi:10.7554/eLife.85998)
Supplement: Figure 7—figure supplement 1—source data 2. [file elife-85998-fig7-figsupp1-data2.pdf]

Figure 7-figure supplement 1A_NCS1

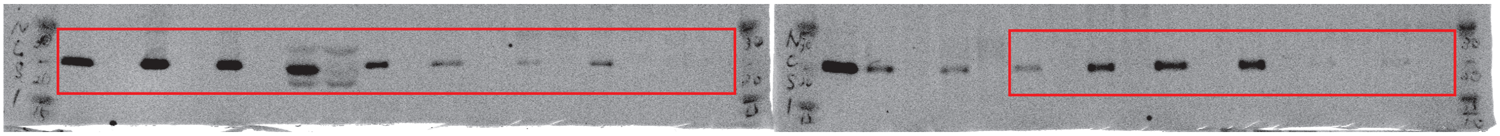

Figure 7-figure supplement 1A_IFT88

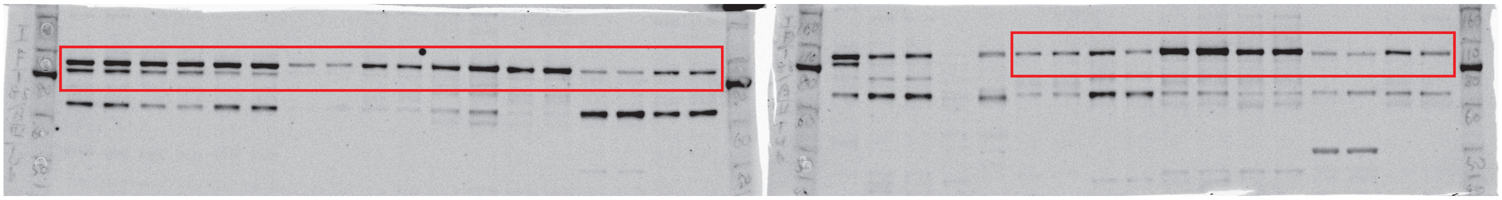

Figure 7-figure supplement 1A_α-Tub

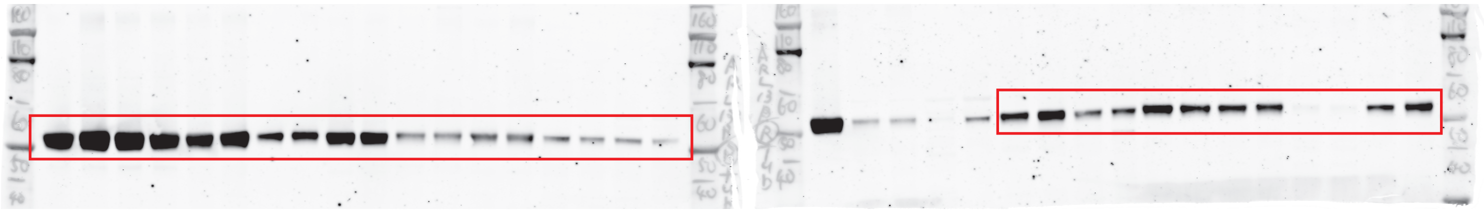

Figure 7-figure supplement 1A_β-TubIII

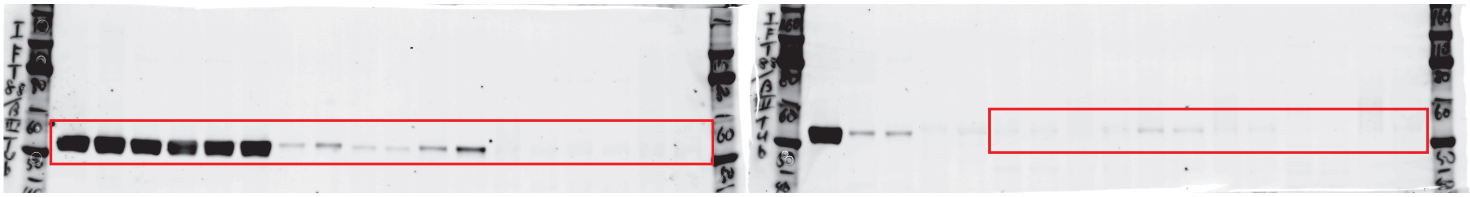

Kanie et al., Figure 7-figure supplement 1-Source Data 2
