# Supplementary material for: Myristoylated Neuronal Calcium Sensor-1 captures the preciliary vesicle at distal appendages
Source: eLife. 2025 Jan 30;14:e85998. doi: 10.7554/eLife.85998 (PMC11984960; doi:10.7554/eLife.85998)

Figure 1D_GFP

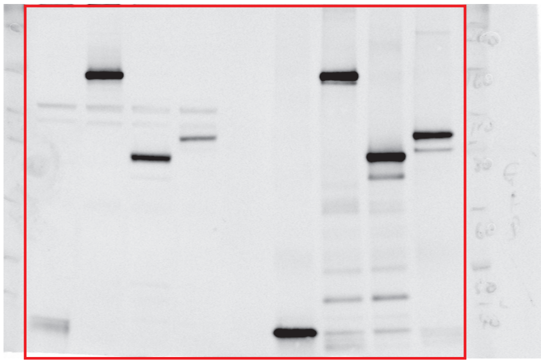

Figure 1D_NCS1

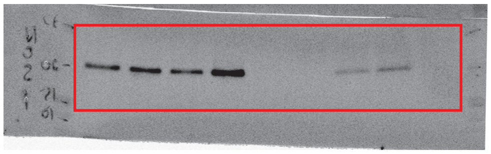

Figure 1D_Tubulin

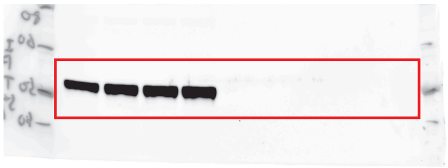

Figure 1E_anti-MYC

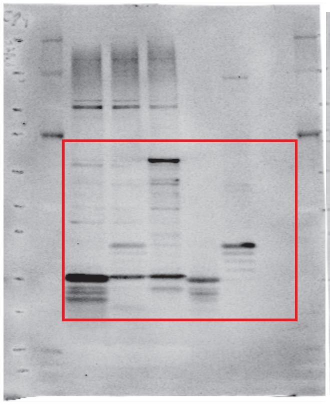

Figure 1E_anti-HA

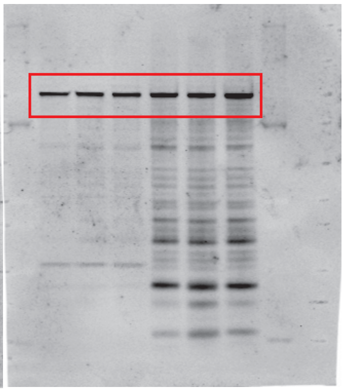

Figure 1F_anti-MYC

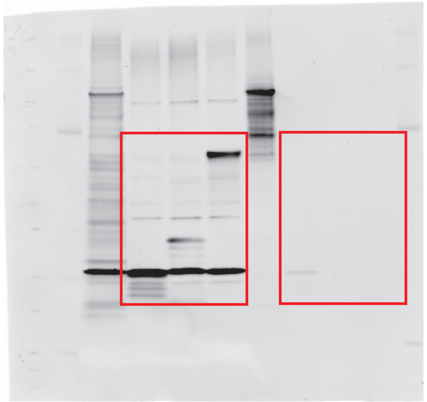

Figure 1F_anti-HA

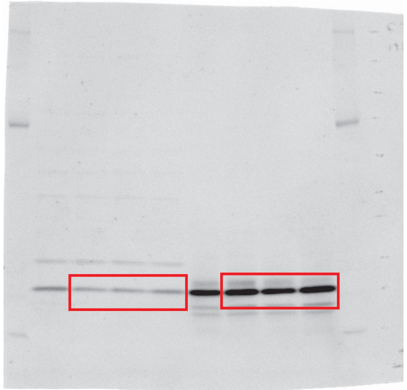

Figure 1-figure supplement1A_CEP89

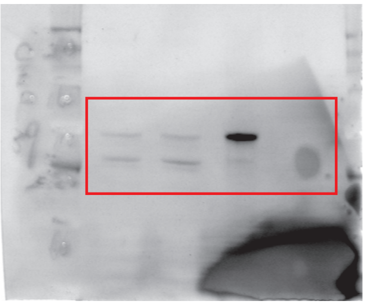

Figure 1-figure supplement1A_NCS1

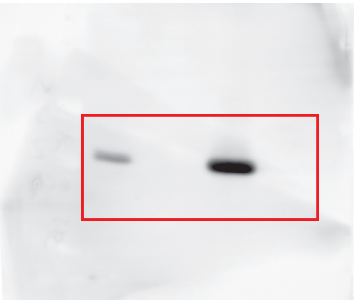

Figure 1-figure supplement 2A_MYC

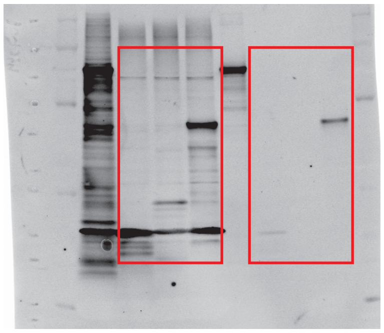

Figure 1-figure supplement 2A_HA

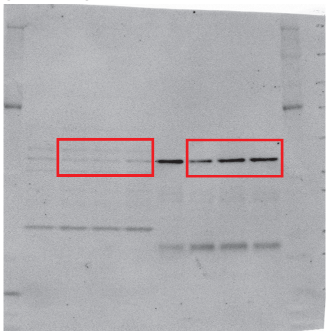

Kanie et al., Resource-full blot

Figure 2-figure supplement 2D_anti-CEP89

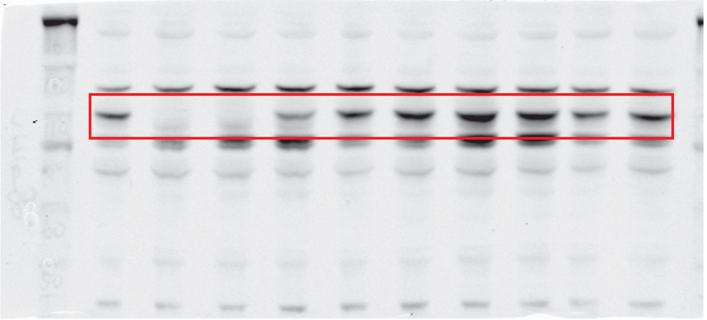

Figure 2-figure supplement 2D_anti-NCS1

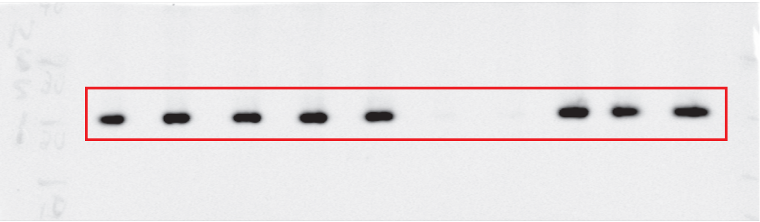

Figure 2-figure supplement 2D_anti-Tubulin

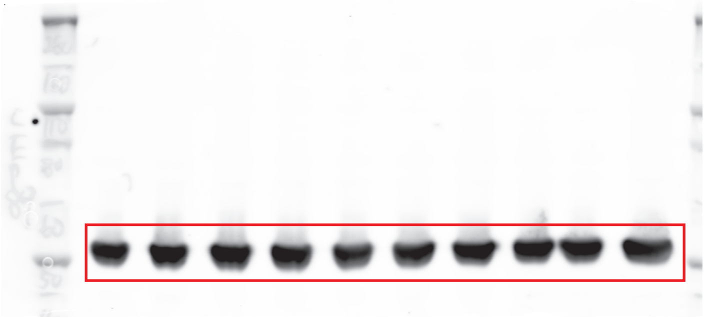

Figure 2-figure supplement 2E_anti-GFP

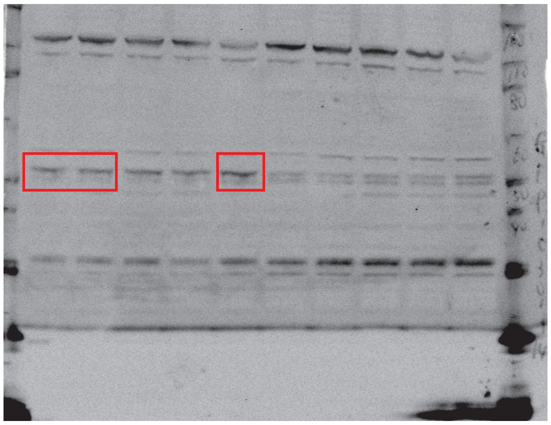

Figure 2-figure supplement 2E_anti-Tubulin

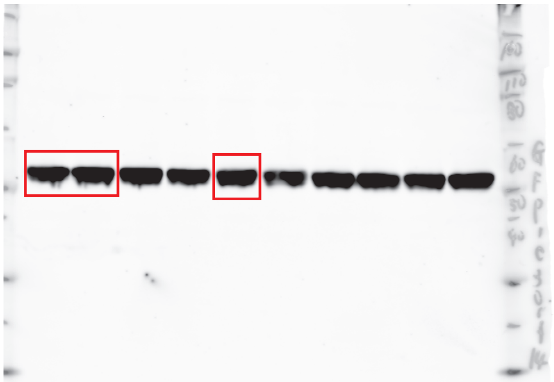

Figure 4A_anti-NCS1

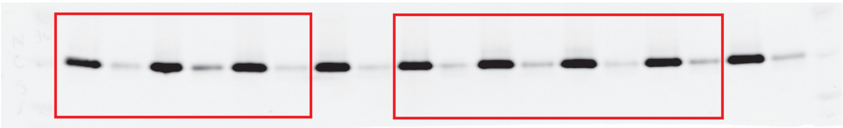

Figure 4A_anti-Tubulin

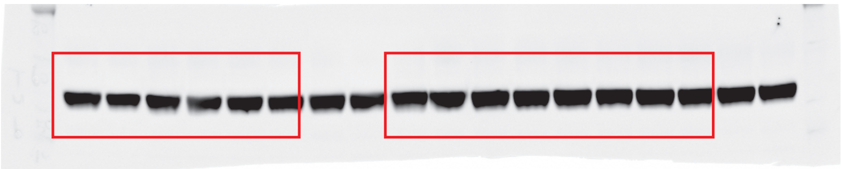

Figure 5A_CEP89

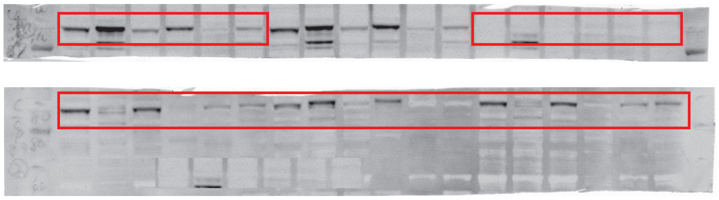

Figure 5A_NCS1

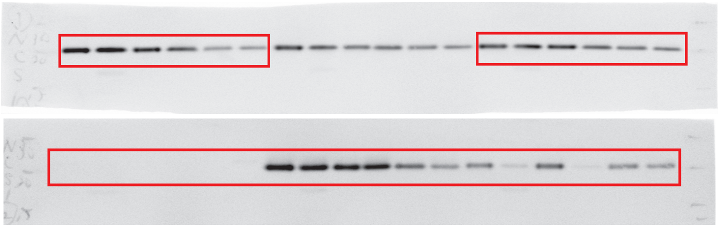

Figure 5A_EGFR

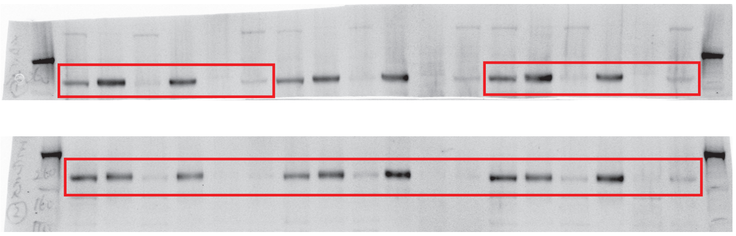

Figure 5A_RabGDI

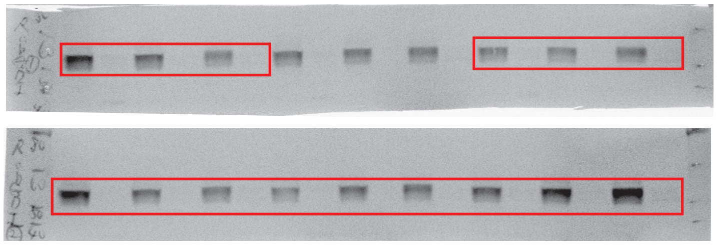

Figure 5B_NCS1

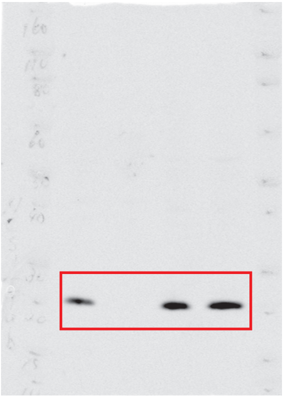

Figure 5B_Tubulin

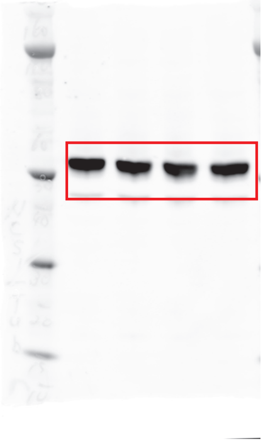

Figure 5-figure supplement 1A_GFP (CEP89)

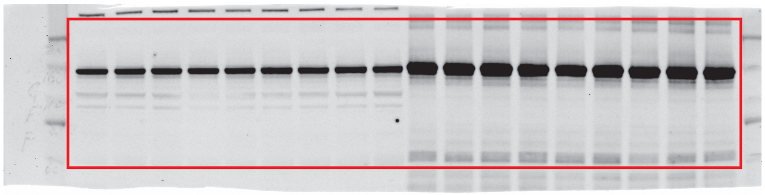

Figure 5-figure supplement 1A_NCS1

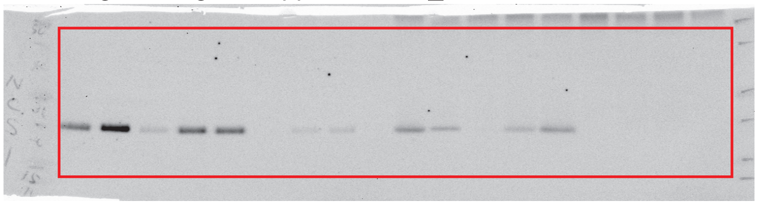

Figure 7-figure supplement 1A_NCS1

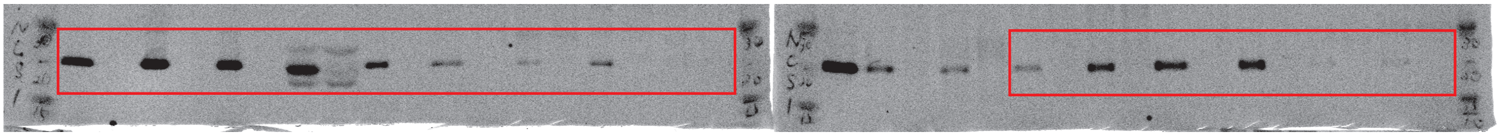

Figure 7-figure supplement 1A_IFT88

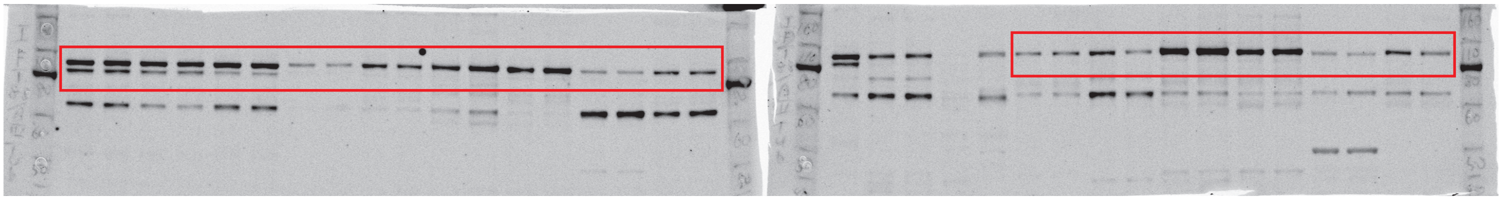

Figure 7-figure supplement 1A_α-Tub

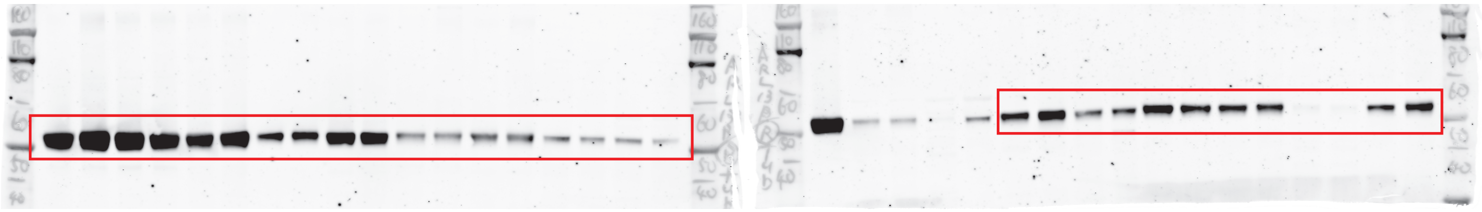

Figure 7-figure supplement 1A_β-TubIII

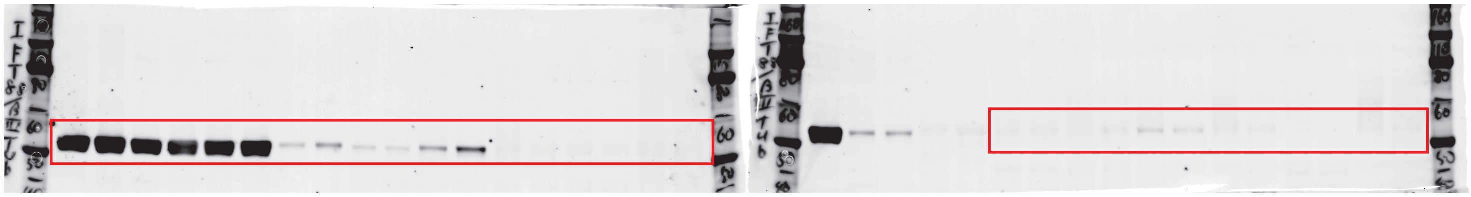

Supplement: Source data 6. [file elife-85998-data6.pdf]
